# Selection and Validation of Reference Genes for Quantitative Real-time PCR in *Gentiana macrophylla*

**DOI:** 10.3389/fpls.2016.00945

**Published:** 2016-06-29

**Authors:** Yihan He, Hailing Yan, Wenping Hua, Yaya Huang, Zhezhi Wang

**Affiliations:** ^1^Key Laboratory of the Ministry of Education for Medicinal Resources and Natural Pharmaceutical Chemistry, National Engineering Laboratory for Resource Development of Endangered Crude Drugs in Northwest of China, College of Life Sciences, Shaanxi Normal UniversityXi’an, China; ^2^School of Geography and Life Science, Qinghai Normal UniversityXining, China; ^3^Department of Life Sciences, Shaanxi XueQian Normal UniversityXi’an, China

**Keywords:** *Gentiana macrophylla*, reference gene, qPCR, transcriptome, gene expression, abiotic stress

## Abstract

Real time quantitative PCR (RT-qPCR or qPCR) has been extensively applied for analyzing gene expression because of its accuracy, sensitivity, and high throughput. However, the unsuitable choice of reference gene(s) can lead to a misinterpretation of results. We evaluated the stability of 10 candidates – five traditional housekeeping genes (*UBC21*, *GAPC2*, *EF-1*α*4*, *UBQ10*, and *UBC10*) and five novel genes (*SAND1*, *FBOX*, *PTB1*, *ARP*, and *Expressed1*) – using the transcriptome data of *Gentiana macrophylla*. Common statistical algorithms Δ*C*_t_, GeNorm, NormFinder, and BestKeeper were run with samples collected from plants under various experimental conditions. For normalizing expression levels from tissues at different developmental stages, *GAPC2* and *UBC21* had the highest rankings. Both *SAND1* and *GAPC2* proved to be the optimal reference genes for roots from plants exposed to abiotic stresses while *EF-1*α*4* and *SAND1* were optimal when examining expression data from the leaves of stressed plants. Based on a comprehensive ranking of stability under different experimental conditions, we recommend that *SAND1* and *EF-1*α*4* are the most suitable overall. In this study, to find a suitable reference gene and its real-time PCR assay for *G. macrophylla* DNA content quantification, we evaluated three target genes including *WRKY30*, *G10H*, and *SLS*, through qualitative and absolute quantitative PCR with leaves under elicitors stressed experimental conditions. Arbitrary use of reference genes without previous evaluation can lead to a misinterpretation of the data. Our results will benefit future research on the expression of genes related to secoiridoid biosynthesis in this species under different experimental conditions.

## Introduction

*Gentiana macrophylla* Pall. is a well-known medicinal plant in the Gentianaceae family. Its dried roots, ‘Qinjiao,’ have been used in traditional Chinese medicine for over 2000 years, usually as an ingredient in numerous formulae. The biological and pharmacological effects of Qinjiao include stomachic, choleretic, and antihepatotoxic activities (Wang and Lou, 1987; Ji et al., 2002). Secoiridoids are its dominant active constituents, especially gentiopicrin (gentiopicroside). With the development of molecular biology tools, *Gentiana* is now being used to study the molecular pathways of secondary metabolites and key related genes.

Improving our understanding of gene expression patterns can provide insight into complex biological processes, such as signaling and metabolic pathways (Marino et al., 2008). Quantitative real-time PCR (qPCR) is the most sensitive method for detecting both high and low levels of expression. This technique is used for clinical diagnoses, analyses of gene expression in specific tissues, and research projects that involve complex experiments and a large number of genes (Gachon et al., 2004; Nicot et al., 2005). There are mainly two kinds of qPCR assays in use: relative quantification and absolute quantification. Relative quantification compares expression of the target gene to that of one or more reference genes within the same sample. Reference genes should be consistently expressed across the samples being surveyed (Sellars et al., 2007). Absolute quantification determines the exact copy concentration of target gene by relating the cycle threshold (*Ct*) value to a standard curve (Yu et al., 2005). This method can accurately quantify the number of template copies in a known amount of starting sample. A set of guidelines, the *M*inimum *I*nformation for Publication of *Q*uantitative Real-time PCR *E*xperiments (MIQE) has been developed to improve the reproducibility and reliability of qPCR experiments (Bustin et al., 2009). Currently in plant research, validated and qualitative RT-qPCR protocols are still rare. Keyser et al. (2013) build the protocol can be implemented on all plant species to assure accurate quantification of gene expression.

Classic housekeeping genes that encode *18S rRNA*, *ubiquitin*, *actin*, β*-tubulin*, and *glyceraldehyde-3-phosphate dehydrogenase* (*GAPC* or *GAPDH*) are commonly used as internal controls for such analyses of plants. However, those genes were chosen in the pre-genomic era because of their known or suspected roles in basic cellular processes. Although they were assumed to have uniform expression in all types of samples and under all experimental conditions (Czechowski et al., 2005), more recent examinations have demonstrated that, for many species and treatments, the expression of these genes is, in fact, highly variable in different testing environments (Czechowski et al., 2005; Jain et al., 2006). In contrast, several new reference genes with very stable expression have been identified through microarray, transcriptome, and genome-wide sequencing analyses that have focused on a range of species, e.g., humans, *Escherichia coli*, and *Arabidopsis thaliana* (hereafter, *Arabidopsis*; Czechowski et al., 2005; Maccoux et al., 2007; Zhou et al., 2011). Furthermore, the rapid introduction of genomes and transcriptome datasets has provided a high-throughput approach for identifying sets of novel reference genes (Zhuang et al., 2015). For example, 40 candidates have been mined from datasets for the *Brassica napus* transcriptome, and 14 have been selected for further qPCR analysis with different tissues and under various experimental treatments (Wang et al., 2014). Expressed sequence tag (EST) databases have been screened to find three novel reference genes and eight traditional housekeeping genes that are stably expressed in different tissues/organs and developing seeds from four cultivars of *Vernicia fordii* (Han et al., 2012). Transcriptome sequence data in *Fagopyrum esculentum* have revealed that *Expressed protein of unknown function* (*Expressed1* or *Exp1*), *SAND family protein* (*SAND*), and *clathrin adapter complex subunit family protein* (*CACS*) are the most stably expressed genes in different structures of that plant (Demidenko et al., 2011). All of these reports demonstrate the importance of screening and identifying novel reference genes from EST databases, transcriptome data, microarray analysis, and cDNA libraries (Kumar et al., 2011). The success of qPCR analyses with *G. macrophylla* is still limited because of inappropriate choices made for reference genes. Identifying more reliable genes to use with that method would benefit future transcription-level studies of *G. macrophylla* development and metabolic pathways, such as for secoiridoid biosynthesis. Transcriptome profiling has been performed for genes expressed in the roots, leaves, and floral parts of this species, and numerous unigenes have been assigned to secondary-metabolite pathways (Hua et al., 2014). This provides a wealth of resources for our screening reference genes.

The object of the research described here was to characterize genes that might be suitable for transcript normalization in *G. macrophylla* plants at different developmental stages or when subjected to abiotic stresses. Expression profiles for 10 candidate genes *SAND1*, *F-box family protein* (*FBOX*), *Ubiquitin-conjugating enzyme 21* (*UBC21*), *Polypyrimidine tract-binding protein 1* (*PTB1*), *GAPC2*, *Actin-related protein* (*ARP*), *Elongation factor 1-alpha 4* (*EF-1*α*4*), *Polyubiquitin 10* (*UBQ10*), *Ubiquitin-conjugating enzyme 10* (*UBC10*), and *Exp1* – were examined in leaves and roots from plants (1-year-old seedlings) exposed to elicitor stress inducers (silver nitrate, copper sulfate, arachidonic acid, ammonium citrate, salicylic acid, or methyl jasmonate); roots, leaves, and stems collected at 1-year-old seedlings; or whole plants sampled at the two-leaf, four-leaf, or six-leaf stage of development. The stability of expression for these genes was then evaluated by the GeNorm (Vandesompele et al., 2002), NormFinder (Andersen et al., 2004), BestKeeper (Pfaﬄ et al., 2004), and comparative Δ*C*_t_ methods (Silver et al., 2006).

In this study, the validity of using the two housekeeping genes – *SAND1* and *EF-1*α*4* – as reference genes to normalize qPCR gene expression data from the *G. macrophylla* is tested. Expression levels of *WRKY transcription factor* (*WRKY30*), *geraniol 10-hydroxylase* (*G10H*), and *secologanin synthases* (*SLS*) gene are determined in a sample set of leaves from plants undergoing abiotic stress. Finally, the direct comparison of the expression profiles by using relative and absolute qPCR procedures enables us to determine if consistent results can be achieved. As transcription factors, the WRKY proteins are involved in responses to biotic and abiotic stresses, and in developmental processes (Ulker and Somssich, 2004). Our digital expression (DGE) database showed that the members of WRKY family from *G. macrophylla* can positively response the elicitors stress (Hua et al., 2014). Secoiridoids, such as gentiopicroside in *G. macrophylla*, are derived from secologanin, which originates from isopentenyl diphosphate via the iridoid pathway (van der Fits and Memelink, 2000). Its biosynthesis in plants might involve either the cytosolic mevalonic acid (MVA) or the plastidial 2-C-methyl-D-erythritol-4-phosphate (MEP) pathway for isopentenyl diphosphate (IPP) and the iridoid pathway for secologanin. Several genes encoding key enzymes in those pathways have been well studied in *Catharanthus roseus* (Hedhili et al., 2007). *G10H* and *SLS* have important roles in regulating monoterpenoid indole alkaloids (MIA) biosynthesis in *C. roseus* (Zhu et al., 2014; Bernonville et al., 2015). Taken together, the aims of this study are (i) to select appropriate reference genes to use for normalization of gene expression by qPCR in *G. macrophylla*, (ii) to develop and evaluate qPCR methods for these genes in medicine plant which have transcriptome data and (iii) will help further efforts to quantify DNA content or copy number, contributing to the advance of *G. macrophylla* molecular pathways of secondary metabolites.

## Materials and Methods

### Plant Materials and Experimental Conditions

Seeds of *Gentiana macrophylla* collected from Taibai County, Shaanxi Province, China, were soaked overnight in running tap water. After sonicated for 30 min and 24 h of gibberellin treatment, they were scattered on soil and germinated in the greenhouse (20 ± 2°C, natural lighting). The roots, stems, and leaves were sampled from 1-year-old (6- to 7-cm-tall) plants. Whole plant tissues were also collected from young seedlings (1- to 2-cm-tall) at the two leaves (2L), four leaves (4L), and six leaves (6L) stages. The effects of abiotic stress on gene expression were monitored by foliage spraies on 1-year-old plants with 0.92 mM AgNO_3_ (Ag), 200 μM CuSO_4_ (Cu), 10 mg L^-1^ arachidonic acid (AA), 200 μM ammonium citrate (AC), 200 μM salicylic acid (SA), or 200 μM methyl jasmonate (MeJA), and samples (root and leaf) were collected separately after 6 h of stress treatment. All tissues tested from each experimental condition were flash-frozen in liquid nitrogen and stored at –80°C.

### Total RNA Extraction and cDNA Synthesis

Total RNA was isolated with a Polysaccharide and Polyphenols Plant Extract Total RNA (centrifugal column type) Kit according to the manufacturer’s instructions (BioTake, Beijing, China). RNA was treated with RNase-free DNase I (TaKaRa, Dalian, China) to remove genomic DNA. The RNA integrity was checked on a 1% agarose gel. The quantity and quality of the total RNA samples were assessed by recording absorbance at 260/280 nm and 260/230 nm with a NanoDrop ND-1000 spectrophotometer (Thermo Scientific, Wilmington, DE, USA). Only RNA samples with a 260/280 ratio of 1.8 to 2.1 and a 260/230 ratio >2.0 were used for subsequent analyses. Total RNA (1 μg) was reverse-transcribed with a PrimeScript^TM^ RT Reagent Kit (TaKaRa, Dalian, China) in a 20-μL reaction volume according to the manufacturer’s protocol. All cDNA samples were diluted at 1:5 with RNase-free water and stored at –80°C.

### Reference Genes Selection and Primer Design

We performed transcriptome sequencing of *G. macrophylla* using Illumina paired-end sequencing technology on an Illumina Hi-Seq^TM^ 2000 platform for the four samples (flowers, stems, leaves, and roots; Hua et al., 2014). To ensure the reliability and correctness of target prediction, we applied two steps to predict the potential reference genes of *G. macrophylla*. The first step was based on *Arabidopsis* sequences used as queries for BLASTn and tBLASTx against the *G. macrophylla* transcriptome, which had been uploaded in the BioEdit (Hall, 1999) local database. Second step, *G. macrophylla* genes used as queries for BLAST one by one through the Tair^1^, the highest *Arabidopsis* ortholog sequences were recorded at **Table 1**. Candidate reference genes of *G. macrophylla* were shown in **Table 1**. The primers were designed according to NCBI Primer-BLAST^2^. Gene characteristics and primer sequences are presented in **Table 1**.

**Table 1:** Candidate reference genes, primer sequences, and characteristics of PCR amplifications in *Gentiana macrophylla*.

Accession No.^a^	Gene	Name	Length (bp)	Primer sequence U/L [5′–3′]	*R*^2^	Slope (-)	*E* (%)	Tm (°C)	Ortholog^b^	Identity (%)
GAJR01001128	SAND1	*SAND family protein*	118	TTC ATG GTG ATT CTC CAG C TTC AAG GAA GAT GAC AAC C	0.99	3.5180	92	80.0	At2g28390	79
GAJR01009142	FBOX	*F-box family protein*	184	CTG GCA TTA TCT GGT GAA G CAA ACT TGG AGG ACG TTA C	1.00	3.5800	90	82.3	At5g15710	80
GAJR01016484	UBC21	*Ubiquitin-conjugating enzyme 21*	142	CCA TCA GAA ACC CCT TAT G GGC AAA TCT CCC CTG TCT	1.00	3.4800	94	83.0	At5g25760	93
GAJR01031250	PTB1	*Polypyrimidine tract-binding protein 1*	210	CAA CAG CGA TAG TAT GGT C CAA GGT TGA TAA CAT ATC CC	1.00	3.7450	85	82.0	At3g01150	63
GAJR01016742	GAPC2	*Glyceraldehyde-3-phosp-hate dehydrogenase C2*	197	AGA ATT GGA CGT TTG GTT G ACT TTG ACA GGC TTC TCA C	1.00	3.5540	91	84.9	At1g13440	89
GAJR01027933	ARP	*Actin-related proteins*	166	GTC TGT GAT AAT GGC ACC G GCA TCT TTA AGC AGG CAT C	0.98	3.5397	92	84.6	At3g27000	88
GAJR01027412	EF-1α4	*Elongation factor 1-alpha*	97	GAC AAG CCT CTG CGT CTC CCA GTT GGT GCA AAG GTG	1.00	3.4093	96	84.4	At1g07930	93
GAJR01041949	UBQ10	*Polyubiquitin 10*	192	TGC TGG TCT GGA ATA C ACG CAC TCT AGC CGA CTA C	1.00	3.4280	96	83.1	At4g05320	99
GAJR01016334	UBC10	*Ubiquitin-conjugating enzyme 10*	162	CAG TAA CGG AAG CAT TTG TTG CCT TTG TCT GTC TTG	1.00	3.9607	79	79.6	At5g53300	96
GAJR01031708	Exp1	*Expressed protein of unknown function*	130	CAG TCT CGG ATG GAC AAT GTT AGG TGG GGT CTT ACC	1.00	3.7170	86	80.8	At4g33380	57

^a^*Gentiana macrophylla* accession numbers; ^b^*Arabidopsis* ortholog locus. *R^*2*^*, coefficient of determination; *E*, PCR amplification efficiency.

### Test Conditions for qPCR and Analysis of Data

Reactions to assess the range of expression for our 10 candidate reference genes were performed in triplicate with SYBR^®^ Premix Ex Taq^TM^II (TaKaRa, USA) and the Roche LightCycler^®^ 96 system (Roche Diagnostics GmbH). Each sample was tested in three independent replicates with a total reaction volume of 25 μL that contained 0.5 μL of each primer (diluted to 10 mM) plus 12.5 μL of SYBR^®^ Premix Ex Taq^TM^ II, 9.5 μL of ddH_2_O, and 2 μL of template. Conditions for qPCR included an initial 95°C for 30 s, then 45 cycles of 95°C for 5 s and 60°C for 30 s, followed by a final melting curve analysis.

To determine how different statistical algorithms compared in their ability to select the most stable reference genes, we applied RefFinder (Xie et al., 2012). This web^3^-based comprehensive tool utilizes the currently available algorithms GeNorm, NormFinder, BestKeeper, and comparative Δ*C*_t_. It assigns an appropriate weight to each gene and calculates a geometric (Geo) mean for overall ranking of all potential reference genes. We used GenEx6 software (MultiD Analysis^4^) to obtain the optimal number of reference genes by calculating their values of Accumulated Standard Deviation (Acc. SD).

### Absolute and Relative Quantitation Method

The *G. macrophylla* transcription factor *WRKY30* and two key enzymes genes (*G10H* and *SLS*) in gentiopicroside pathway were assessed in the present study for ten potential endogenous genes suitability in qPCR. The PCR-amplifications were performed under conditions of 94°C for 2 min; then 30 cycles of 94°C for 30 s, 60°C for 30 s, and 72°C for 30 s; followed by a final extension step at 72°C for 10 min. Each sample with a total reaction volume of 50 μL that contained 1 μL of each primer (diluted to 10 mM) plus 25 μL of Taq PCR master Mix (Takera, Dalian, China), 21 μL of ddH2O, and 2 μL of template. Afterward, 50 μL of each PCR product was run on a 1% agarose gel for confirmation. Amplified products of the correct (predicted) size were excised from the agarose gels and purified with an E.Z.N.A.TM Gel Extraction Kit (OMEGA, USA). All of them were sequencing carried out by Shanghai Biological Engineering Company. All sequencing results were alignment with *G. macrophylla* transcriptome database confirmed to be the expected amplicon.

DNA (PCR product) concentration was estimated by measuring the absorbance at 260 nm as described above. DNA copy number was calculated according to the following formula (Godornes et al., 2007):

$$\begin{array}{l} Numer\;of\;copies(copies/\mu L)=6.02\times 10^{23}(copies/mol)\times \\ \;DNA\;concentrations\;(g/\mu L)/[number\;of\;bases\;pairs\;\times 660\;Daltons] \end{array}$$

6.02 × 10^23^ (molecules/mole) Avogadro’s number

660 Da Average weight of a single base pair.

Each of the purified DNA was diluted with sterile deionized water to obtain a standard series differing by 10-fold.

After qPCR reaction, the values of threshold cycles are achieved. From the slope of a standard curve, PCR amplification efficiency (*E*) can be calculated according to the equation as follow (Leong et al., 2007):

$$E=[10^{\left(-1/slope\right)}-1]\times 100\%$$

Where the “slope” is that of the linear regression of Log (target concentration) versus threshold cycle (*C*_t_; Gonçalves et al., 2005).

Each standard curve is established by plotting the *C*_t_ on the *Y*-axis and the natural log of concentration (copies/μL) on the *X*-axis, and the equation *y* = slope × *x* + *b*, coefficient of determination (*R*^2^) and percentage of variance in copy numbers were achieved (Xue et al., 2014). Primers used for analyzing genes expression, plus information about the standard curves, *R^2^* and *E* are shown in **Table 2**.

**Table 2:** The standard curve formula, coefficient of determination (*R*^2^) and PCR amplification efficiency (*E*) performed in qPCR assays.

Gene	Accession No.	Primer sequence U/L [5′–3′]	Standard curve formula	*R*^2^	*E* (%)
WRKY30	GAJR01036722	CAG ATT CGG AAG CAT GTG A GCA AGT GGT GAT TTT GGA AG	*y* = -3.5933*X*+ 39.25	1.0 0	90
SLS	GAJR01014973	CAC ATT CAC CAT ACC ATC C ACT TCC AAT ACC AGA GAG C	*y* = -3.8760*X* + 39.8	1.0 0	81
G10H	GAJR01006003	ATC ATG GGC TTA CAG TTC G ACA GGG AGC CAA ATA ACA G	*y* = -3.5735*X* + 38.49	0.9 3	90

Relative quantitation analysis of expression data for target gene was conducted according to the 2^-ΔΔ^*^C^*^t^ method (Erickson et al., 2007).

## Results

### PCR Amplification Specificity and PCR Efficiency of Candidate Reference Genes

**FIGURE 1:**
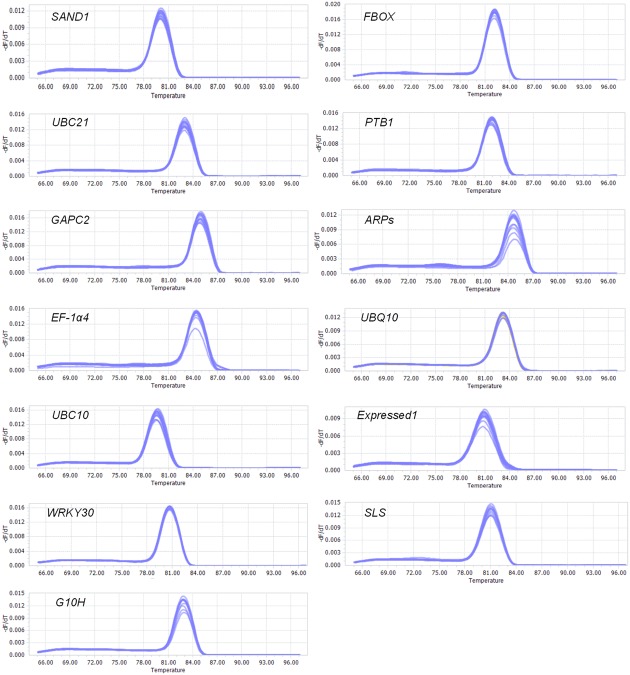
**Melting curves for genes.** Melting temperatures were visualized by plotting negative derivative of change in fluorescence divided by change in temperature relative to temperature [-(d/dT) Fluorescence].

The products of RT-qPCR reactions were sequenced and shown to be identical to the sequence accessions in **Table 1**. Primer specificities (**Table 1**) were confirmed by single-peak melting curves for the qPCR products (**Figure 1**), based on the presence of a single band of the correct size for each pair. The melting temperatures (Tm) of the PCR products ranged from 79.6°C for *UBC10* to 84.9°C for *GAPC2* (**Table 1**). Primers that displayed coefficients of correlation >0.98 and efficiencies between 79 and 94% were selected for the next round of qPCR (**Table 1**).

**FIGURE 2:**
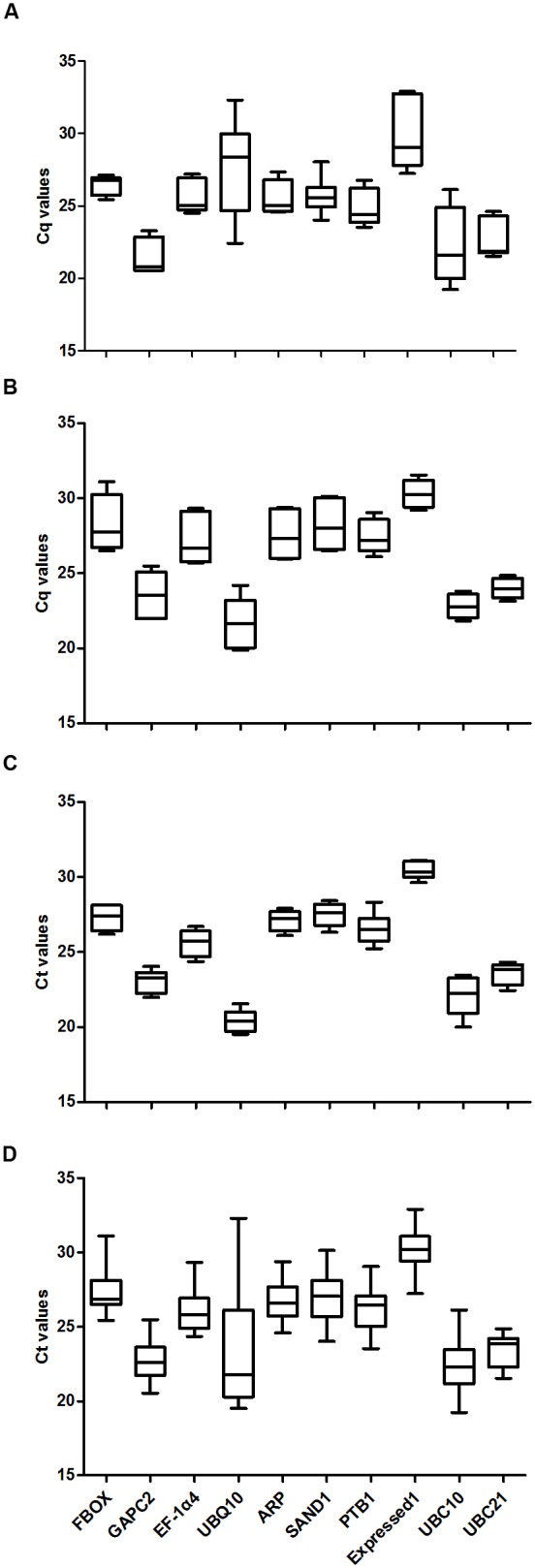
**Average *C*_t_ values for 10 candidate reference genes. (A)** developmental stages, **(B)** leaves from stress-treated plants, **(C)** roots from stress-treated plants, **(D)** Data from all experimental conditions combined.

Data were analyzed for experiments covering either plant developmental stages or abiotic stress responses. Four sample subsets were examined: tissues from various stages, leaves from plants exposed to abiotic stress, roots from those stressed plants, and a combination of data from all experimental conditions (all samples). The expression levels of housekeeping genes and transcript accumulations are shown in **Figure 2**. Transcripts of *UBQ10* were most abundant in roots from stressed plants (median cycle threshold, or *C*_t_, value of 20.4) while those levels were lowest in the sample set for developmental stages (*C*_t_ of 27.6). Expression was low in all four sample subsets for *Exp1*. Overall, *C*_t_ values ranged from 19.22 for *UBC10* in stems to 32.9 for *Exp1* at 4 L. Most of those values were between 22.0 and 28.0. These results indicated that none of the selected genes had stable expression among samples, thereby demonstrating how important it is to evaluate the suitability of reference gene(s) for normalizing expression under given experimental conditions when analyzing *G. macrophylla*.

### Ranking of Candidates and Determination of Optimal Reference Genes

Four algorithms were used to analyze 10 housekeeping genes and four sample subsets across 16 experimental conditions. An integration tool calculated the Geomean of each gene across GeNorm, NormFinder, BestKeeper, and Δ*C*_t_ methods. From this, the M values (gene expression stability value, via GeNorm) and SD values (stability values, NormFinder) are presented in **Table 3**. The reference genes were ranked according to their comprehensive stability in **Table 4**.

**Table 3:** Stability rankings of candidate genes based on GeNorm and NormFinder for four sampling subsets (Acc. SD, accumulated standard deviation).

Leaves from stressed plants					Roots from stressed plants					Developmental stage					All samples				
GeNorm		NormFinder			GeNorm		NormFinder			GeNorm		NormFinder			GeNorm		NormFinder		
Gene	*M*-value	Gene	*SD*	Acc. SD	Gene	*M*-value	Gene	*SD*	Acc. SD	Gene	*M*-value	Gene	*SD*	Acc. SD	Gene	*M*-value	Gene	*SD*	Acc. SD
*SAND1*	0.45	*EF-1*α*4*	0.29	0.29	*SAND1*	0.48	*SAND1*	0.43	0.43	*UBC21*	0.28	*GAPC2*	0.03	0.03	*SAND1*	0.57	*SAND1*	0.50	0.50
*EF-1*α*4*	0.45	*SAND1*	0.40	0.25	*GAPC2*	0.48	*ARP*	0.48	0.32	*GAPC2*	0.28	*UBC21*	0.14	0.07	*EF-1*α*4*	0.57	*UBC21*	0.58	0.38
*UBC10*	0.66	*UBC21*	0.84	0.33	*EF-1*α*4*	0.54	*GAPC2*	0.48	0.27	*ARP*	0.34	*SAND1*	0.27	0.10	*UBC21*	0.74	*EF-1*α*4*	0.59	0.32
*UBC21*	0.87	*UBC10*	0.88	0.33	*ARP*	0.64	*EF-1*α*4*	0.51	0.24	*PTB1*	0.39	*ARP*	0.47	0.14	*ARP*	0.88	*Exp1*	0.94	0.34
*Exp1*	1.02	*Exp1*	1.09	0.34	*UBQ10*	0.75	*UBQ10*	0.67	0.23	*EF-1*α*4*	0.44	*PTB1*	0.58	0.16	*GAPC2*	0.99	*UBC10*	1.08	0.35
GAPC2	1.24	*PTB1*	1.39	0.37	*FBOX*	0.79	*Exp1*	0.68	0.23	*SAND1*	0.52	*EF-1*α*4*	0.74	0.18	*PTB1*	1.11	*ARP*	1.10	0.34
*PTB1*	1.39	*UBQ10*	1.58	0.39	*Exp1*	0.81	*FBOX*	0.70	0.22	*FBOX*	0.68	*Exp1*	1.18	0.23	*Exp1*	1.21	*GAPC2*	1.13	0.33
*UBQ10*	1.50	*ARP*	1.62	0.39	*PTB1*	0.85	*UBC21*	0.89	0.22	*Exp1*	0.87	*UBC10*	1.29	0.26	*UBC10*	1.28	*PTB1*	1.26	0.33
*ARP*	1.58	*GAPC2*	1.68	0.40	*UBC21*	0.91	*PTB1*	1.00	0.23	*UBC10*	1.01	*FBOX*	1.48	0.28	*FBOX*	1.36	*FBOX*	1.42	0.34
*FBOX*	1.70	*FBOX*	1.93	0.41	*UBC10*	1.02	*UBC10*	1.35	0.24	*UBQ10*	1.39	*UBQ10*	3.13	0.40	*UBQ10*	1.88	*UBQ10*	4.31	0.53

**Table 4:** Stability rankings by RefFinder of candidate reference genes from four sampling subsets representing different experimental conditions.

	Leaves from stressed plants					Roots from stressed plants					
Ranking A	Δ*C*t	BestKeeper	NormFinder	GeNorm	Ranking B	Δ*C*t	BestKeeper	NormFinder	GeNorm	Ranking B
1	*EF-1*α*4*	*EF-1*α*4*	*EF-1*α*4*	*EF-1*α*4 | SAND1*	*EF-1*α*4*	*SAND1*	*Exp1*	*SAND1*	*GAPC2 | SAND1*	*SAND1*
2	*SAND1*	*SAND1*	*SAND1*		*SAND1*	*ARP*	*EF-1*α*4*	*ARP*		*GAPC2*
3	*UBC21*	*UBC21*	*UBC21*	*UBC10*	*UBC21*	*GAPC2*	*SAND1*	*GAPC2*	*EF-1*α*4*	*ARP*
4	*UBC10*	*UBC10*	*UBC10*	*UBC21*	*UBC10*	*EF-1*α*4*	*ARP*	*EF-1*α*4*	*ARP*	*EF-1*α*4*
5	*Exp1*	*Exp1*	*Exp1*	*Exp1*	*Exp1*	*UBQ10*	*UBQ10*	*UBQ10*	*UBQ10*	*Exp1*
6	*PTB1*	*PTB1*	*PTB1*	*GAPC2*	*PTB1*	*Exp1*	*GAPC2*	*Exp1*	*FBOX*	*UBQ10*
7	*ARP*	*UBQ10*	*UBQ10*	*PTB1*	*UBQ10*	*FBOX*	*UBC21*	*FBOX*	*Exp1*	*FBOX*
8	*UBQ10*	*GAPC2*	*ARP*	*UBQ10*	*GAPC2*	*UBC21*	*FBOX*	*UBC21*	*PTB1*	*UBC21*
9	*GAPC2*	*ARP*	*GAPC2*	*ARP*	*ARP*	*PTB1*	*PTB1*	*PTB1*	*UBC21*	*PTB1*
10	*FBOX*	*FBOX*	*FBOX*	*FBOX*	*FBOX*	*UBC10*	*UBC10*	*UBC10*	*UBC10*	*UBC10*

	**Developmental stage**					**All samples**				
**Ranking A**	**Δ*C*t**	**BestKeeper**	**NormFinder**	**GeNorm**	**Ranking B**	**Δ*C*t**	**BestKeeper**	**NormFinder**	**GeNorm**	**Ranking B**

1	*UBC21*	*FBOX*	*GAPC2*	*GAPC2 | UBC21*	*GAPC2*	*SAND1*	*EF-1*α*4*	*SAND1*	*EF-1*α*4 | SAND1*	*SAND1*
2	*GAPC2*	*ARP*	*UBC21*		*UBC21*	*EF-1*α*4*	*UBC21*	*UBC21*		*EF-1*α*4*
3	*ARP*	*PTB1*	*SAND1*	*ARP*	*ARP*	*UBC21*	*SAND1*	*EF-1*α*4*	*UBC21*	*UBC21*
4	*PTB1*	*GAPC2*	*ARP*	*PTB1*	*PTB1*	*ARP*	*FBOX*	*Exp1*	*ARP*	*ARP*
5	*SAND1*	*EF-1*α*4*	*PTB1*	*EF-1*α*4*	*FBOX*	*GAPC2*	*Exp1*	*UBC10*	*GAPC2*	*Exp1*
6	*EF-1*α*4*	*UBC21*	*EF-1*α*4*	*SAND1*	*SAND1*	*Exp1*	*ARP*	*ARP*	*PTB1*	*GAPC2*
7	*Exp1*	*SAND1*	*Exp1*	*FBOX*	*EF-1*α*4*	*PTB1*	*GAPC2*	*GAPC2*	*Exp1*	*PTB1*
8	*UBC10*	*Exp1*	*UBC10*	*Exp1*	*Exp1*	*UBC10*	*PTB1*	*PTB1*	*UBC10*	*UBC10*
9	*FBOX*	*UBC10*	*FBOX*	*UBC10*	*UBC10*	*FBOX*	*UBC10*	*FBOX*	*FBOX*	*FBOX*
10	*UBQ10*	*UBQ10*	*UBQ10*	*UBQ10*	*UBQ10*	*UBQ10*	*UBQ10*	*UBQ10*	*UBQ10*	*UBQ10*

*(Ranking A: 1–10, most to least stable; Ranking B: recommended comprehensive ranking).*

When all samples were considered, the best genes for RT-qPCR normalization in *G. macrophylla* were *EF-1*α*4* (*M* = 0.57, *SD* = 0.59), *UBC21* (*M* = 0.74, *SD* = 0.58), and *SAND1* (*M* = 0.57, *SD* = 0.50). The least suitable overall were *FBOX* (*M* = 1.36, *SD* = 1.42) and *UBQ10* (*M* = 1.88, *SD* = 4.31). However, those rankings changed when the samples were classified into three different groups, i.e., leaves from stressed plants, roots from stressed plants, and developmental stage. The data subset for leaves from stressed plants indicated that the most stable reference genes were *EF-1*α*4* (*M* = 0.45, *SD* = 0.29) and *SAND1* (*M* = 0. 45, *SD* = 0.40) while the least stable were *ARP* (*M* = 1.58, *SD* = 1.62) and *FBOX* (*M* = 1.70, *SD* = 1.93).

For the group of developmental stage samples, the first choice for most accurate normalization was *UBC21* (*M* = 0.28, *SD* = 0.14), followed by *GAPC2* (*M* = 0.28, *SD* = 0.03). In contrast, *UBC10* (*M* = 1.01, *SD* = 1.29) and *UBQ10* (*M* = 1.39, *SD* = 3.13) were the least suitable for use as references (**Table 3**). For roots from stressed plants, the best choices were *SAND1* (*M* = 0.48, from GeNorm; *SD* = 0.43, from NormFinder), *GAPC2* (*M* = 0.48; GeNorm), and *ARP* (*SD* = 0.48; NormFinder). These findings again provided evidence that reference genes must be carefully selected to match the experimental conditions under which a gene is being evaluated.

**FIGURE 3:**
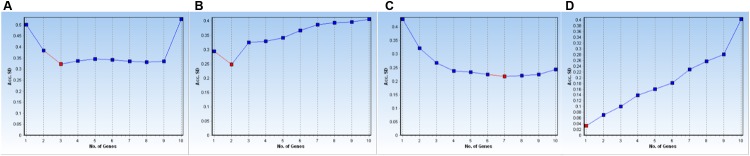
**Determination of optimal number of reference genes for normalization based on Acc. SD calculations.** Data were analyzed by NormFinder for various data subsets of experimental conditions: **(A)** all samples, **(B)** leaves from stressed plants, **(C)** roots from stressed plants, and **(D)** samples from developmental stages.

The GeNorm and NormFinder algorithms available in the GenEx package also allow one to determine the optimal number of control genes to use in normalization processes based on calculations of Acc. SD values for all 10 genes under every experimental condition were computed here by NormFinder and the tested data are shown in **Table 3**. When three reference genes were used for the subset that combined all samples, the lowest Acc. SD value was achieved, i.e., 0.3231 (**Figure 3A**). For leaves from stressed plants, the optimal number of reference genes was two, for a minimum Acc. SD value of 0.2481 (**Figure 3B**). By comparison, evaluations of expression in roots from stressed plants were most accurate when six (Acc. SD value of 0.6390) to seven (0.7518) reference genes were used (**Figure 3C**). Finally, we found it most remarkable that only one gene was needed to provide qPCR normalization for genes from samples in the developmental stage subset (**Figure 3D**).

To obtain comprehensive rankings of these genes as suitable references, we utilized RefFinder and integrated the results from our four algorithms. As shown in **Table 3**, *EF-1*α*4* and *SAND1* (leaves), *SAND1* and *GAPC2* (roots), *GAPC2* and *UBC21* (developmental stages), and *SAND1* and *EF-1*α*4* (all samples) were the most stable while the least appropriate were *ARP* and *FBOX* (leaves), *PTB1* and *UBC10* (roots), *UBC10* and *UBQ10* (developmental stages), and *FBOX* and *UBQ10* (all samples).

### Validation of Selected Reference Genes

Here, the validity of using the two stable genes, *SAND1* and *EF-1*α*4*, as reference genes to normalize real-time RT-PCR gene expression data from the *G. macrophylla* was tested. Expression patterns of three target genes (*WRKY30*, *G10H*, and *SLS*) in a sample set of leaves from stressed plants and mRNAs were quantified using relative and absolute real-time RT-PCR procedures. Standard curves of the above three target genes primer pairs were established, respectively, to evaluate the amplification efficiency, and melting curves were used to check the within-species-specificity of each qPCR reactions (**Figure 1**). The data in **Table 2** indicated that the *R*^2^ values of the three primer sets for the standard curves were >0.98 and the estimated amplification efficiencies (*E*) were between 81 and 90%.

**FIGURE 4:**
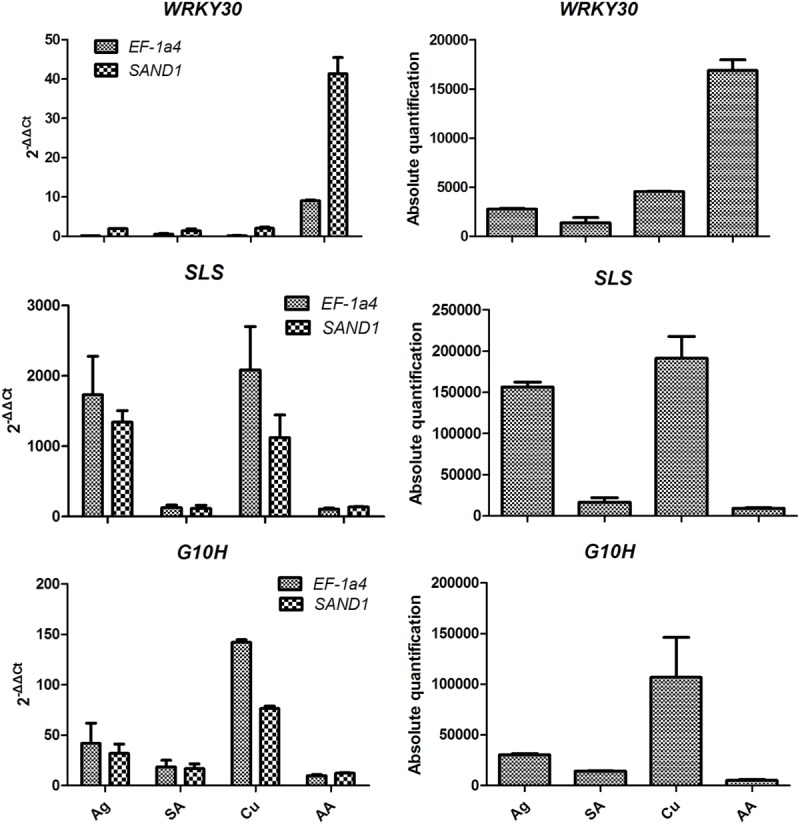
**Transcript levels of WRKY30 transcription factor and two key enzyme genes (*SLS* and *G10H*) in *Gentiana macrophylla* when selected reference genes were used for normalization.** Error bars shows mean standard error calculated from two biological replicates. Comparison of target genes expression profiles relative to the two most stable reference genes (*EF-1a4* and *SAND1*) in samples with leaves from stressed plants. Ag, silver nitrate; SA, salicylic acid; Cu, copper; AA, arachidonic acid.

The expression of *WRKY30* (*P* < 0.001) significantly up-regulated treatment with AA when normalization with *SAND1* or *EF-1*α*4*. The expression of *G10H* was sharply increased at Cu but was relatively lower in the AA and SA stress. Normalization with the most stable genes indicated that *SLS* expression was down-regulated in SA and AA samples when compared with Ag and Cu samples. When target gene expression calculated using relative quantification was more similar to that of the absolute procedure when the stable reference genes were used (**Figure 4**).

## Discussion

With the rapid development of next-generation sequencing technology, RNA sequencing (RNA-Seq) has been applied primarily to analyze the transcriptomes of various species. Although the main outcome has been the identification of differentially expressed genes, these RNA-Seq data are also used to search for reference genes (Zhuang et al., 2015). We previously examined large-scale transcriptome data for *G. macrophylla* that comprised 42,918 unigenes (Hua et al., 2014). That initial search served as our resource for selecting reference genes. For any species, it is crucial that one carefully choose the most stable reference gene or internal control gene in order to avoid misinterpreting the results from expression analyses. In the research presented here, we examined four groups of homologous genes (*EF-1*α, *GAPC*, *ACT*, and *SAND*) and investigated their levels of expression under different experimental conditions. The genes with the highest rankings overall for stable expression were *SAND1* and *GAPC2*. We also chose eight other reference genes, including four traditional housekeeping genes (*UBC21*, *EF-1*α*4*, *UBQ10*, and *UBC10*) and four novel genes (*FBOX*, *PTB1*, *ARP*, and *Exp1*) and compared their levels of expression with those of orthologs from *Arabidopsis*. These 10 reference genes were evaluated in different tissues from *G*. *macrophylla* and under several types of abiotic stress. Our findings demonstrated that RNA-Seq data sets are useful resources when screening suitable candidates, and our results represent an important strategy for large-scale selection of reference genes when studying a non-model plant species (Zhuang et al., 2015).

The statistical algorithms GeNorm, NormFinder, and BestKeeper were developed as aids in selecting stably expressed reference genes for use with qPCR when normalizing expression. For GeNorm, an optimal number of reference genes was selected out of a larger group of candidates based on *M*-values. In contrast, NormFinder evaluates the expression stability of individual reference genes and takes into account intra- and intergroup variations for normalization while BestKeeper analyzes variabilities in the expression of candidate reference genes by calculating fluctuations in *C*_t_ values. All three methods utilize different strategies that can produce contrasting results (Mallona et al., 2010; Wang et al., 2014). For example, in *Arabidopsis*, *ACT2*, *EF-1a4*, and *UBQ10* are ranked immediately after the top three reference genes identified via GeNorm but are ranked lower by NormFinder (Remans et al., 2008). In the current research, *FBOX* proved to be most stably expressed according to BestKeeper but was ranked ninth by GeNorm and seventh by NormFinder. Instead, the Δ*C*_t_, NormFinder, and GeNorm methods recommended *GAPC2* and *UBC21* as most appropriate for normalizing expression during developmental stages. For roots from stressed plants, the top three reference genes were *SAND1*, *ARP*, and *GAPC2* per GeNorm and Δ*C*_t_ while BestKeeper ranked *SAND1* in third place, *ARP* in fourth, and *GAPC2* as sixth for the same tissue. When the data subset for all samples was studied, *EF-1a4* was recognized as the most stably expressed by GeNorm but was ranked third by NormFinder. None of these algorithms identified a single gene as being the most stably expressed under all of our experimental conditions, and individual rankings for each gene differed among algorithms (Klie and Debener, 2011). Therefore, the results from all four methods be considered together when determining which reference genes are most suitable for qPCR normalizations (Wang et al., 2014).

The ideal reference gene shows a constant level of expression that does not vary by organ or tissue type and is also not influenced by the treatment that is applied (Remans et al., 2008). However, numerous studies have shown that no gene is always permanently and stably expressed. Therefore, reference genes must be evaluated for each plant species and for each experimental setup (Hruz et al., 2011). Our results here indicated that, when performing expression analysis with genes from *G*. *macrophylla*, *SAND1*/*EF-1*α*4* are the most appropriate for all samples combined; *EF-1a4*/*SAND1* and *SAND1*/*GAPC2* are the most stably expressed gene pairs in leaves and roots, respectively, from stressed plants; and *GAPC2*/*UBC21* should be used as reference genes when examining expression during various developmental stages.

Stürzenbaum and Kille (2001) and Dean et al. (2002) have stipulated that *EF-1*α is a good invariant control. Earlier studies with *Chrysanthemum* and *Caragana intermedia* also showed that this elongation factor is the most stable reference gene for leaf tissue under stress treatment (Gu et al., 2011; Zhu et al., 2013). Similarly, we found here that *EF-1*α*4* was more stably expressed in leaves from stressed plants but was less stably expressed in roots from stressed plants or in tissues at various developmental stages.

The SAND family protein is involved in vacuolar fusion at the tethering/docking stage in yeast (Wang et al., 2003) and also participates in endosomal traffic in *Caenorhabditis elegans* (Poteryaev et al., 2007). Our examination of stability revealed that *SAND1* was the most stable reference gene in the all-sample data subset as well as in roots and leaves from stressed plants. It also proved to be a better candidate internal control gene in *G. macrophylla*. In *Caragana intermedia*, *SAND* exhibits stable expression across an assortment of tissues under different abiotic stress conditions (Zhu et al., 2013). Furthermore, this gene is one of the most stably expressed in different tissues and organs of citrus genotypes (Mafra et al., 2012).

Ubiquitin conjugation is a protein modification that occurs in a multistep reaction. It sequentially involves an E1 enzyme (ubiquitin-activating enzyme), an E2 enzyme (ubiquitin-conjugating), and an E3 enzyme (ubiquitin ligase). Both *UBC21* and *UBC10* are in the E2 class (Vierstra, 2003; Kraft et al., 2005). In all four of our data subsets, *UBC21* was more stable than *UBC10*. In citrus under viral stress, the pairing of *UBC21*/*UPL7* is the most stable, followed by *UBC9* (Mafra et al., 2012). *UBC21* has been shown to be stably expressed in sample sets of *Arabidopsis* (Czechowski et al., 2005). Finally, *UBC10* in *Cocos nucifera* is a stable reference gene for all stress treatments and endosperm developmental stages (Xia et al., 2014).

Our overall rankings placed *ARP*, *Exp1*, and *GAPC2* at the fourth, fifth, and sixth positions when all samples were considered. The actin-related proteins are members of an actin family that accumulate in the nucleus (Weber et al., 1995; Harata et al., 2000). Some ARPs are clearly involved in cytoskeletal functions. This is based on two related models in which actin and/or *ARPs* function as conformational switches that control either the activity or the assembly of chromatin-remodeling machines (Boyer and Peterson, 2000). To the best of our knowledge, *ARPs* have not previously been used as reference genes for accurate normalization of gene expression data. However, *ARP* ranked third or fourth for our other data subsets except for leaves from stressed plants. That performance was much better than *UBQ10*, *UBC10*, and *FBOX*, which have traditionally been used as stable reference genes in many plants. *GAPDH* (*GAPC*) encodes a glycolytic enzyme that commonly serves as an internal control (albeit without testing) across different species (Li et al., 2012). In our results, *GAPC2* ranked eighth for all samples combined but was ranked first for developmental stages, sixth for leaves from stressed plants, and second for roots from stressed plants. By contrast, this gene has been reported as very unstable in the buds, seeds, and various other organs of leafy spurge (*Euphorbia esula*; Chao et al., 2012).

As stable housekeeping genes in several plant species (Jain et al., 2006), including *Arabidopsis thaliana* (Hanna et al., 2010), *UBQ*s function in response to adverse environments (Fort et al., 1985; Bhatia et al., 1994), making them atypical for those roles. For example, expression of four *UBQ* genes is significantly changed in different tissues and is especially high in the flowers and fruits of *Citrus japonica* (Hu et al., 2014). Similar to our findings here, *UBQ10* shows unstable expression across sample pools for *Glycine max* (Jian et al., 2008) and *Oryza sativa* (Jain et al., 2006).

An ortholog of At4g33380, *Exp1* was moderately stable in our expression analysis. That gene is a good reference in *Arabidopsis* (Czechowski et al., 2005). Another very stable gene in *Arabidopsis*, *FBOX*, is highly expressed in the roots and shoots in response to Cd and Cu treatments (Remans et al., 2008). However, expression of this gene is quite unstable in seeds of leafy spurge during the germination phase (Chao et al., 2012). We also found that *FBOX* was one of the least stable candidate gene under the conditions and subsets tested here. Therefore, all of these reports demonstrate again that the expression of these 10 candidate reference genes can be species-specific and can also vary according to the experimental environment.

The traditional Chinese medicine of the plant *G. macrophylla* is derived from secoiridoid active compounds, especially gentiopicroside, which are abundant in these plants, and have broad biological and pharmacological effects. Elicitors have been used to increase the production or to induce *de novo* synthesis of secondary metabolites in plants. Such treatment could lead to substantial changes in the cellular metabolism (Moreno et al., 1996). The activities of several key enzymes always involved in the biosynthesis of secondary metabolite. Finally, two enzymes genes (*G10H* and *SLS*) and one transcription factor gene (*WRKY30*) were used to confirm the suitability of the reference genes identified here. The biosynthesis of secologanin consists of a number of steps in which the first committed step is the hydroxylation of geraniol to 10-hydroxygeraniol by the enzyme G10H (Moreno et al., 1996). The loganic acid is converted to secologanin via *SLS* (Pan et al., 2015). The *WRKY* genes function involved in developmental processes as well as plant responses to biotic and abiotic stresses (Ulker and Somssich, 2004). In our *G. macrophylla* leaves, When *SAND1* was used as a reference, target gene expression was more similar to that of the absolute method than when *EF-1*α*4* was used as a reference. This study indicates that the use of *SAND1* and *EF-1*α*4* for studying relative gene expression patterns in *G. macrophylla* elicitor stressed samples will give appropriate results. Therefore, it is possible that the novel reference gene identified here can outperform commonly used housekeeping genes. This provided more evidence that the incorrect use of reference genes without validation can reduce precision or produce misleading results.

Based on the outcome of our evaluation, we conclude that *SAND1* and *EF-1*α*4* is the most appropriate reference gene for expression analysis when tissue types under various abiotic stress conditions. Our results also demonstrate that no gene can act as a universal reference and they highlight the importance of systematically examining expression under each set of experimental conditions (Gutierrez et al., 2008). We also identified novel reference genes that outperform the housekeeping genes commonly used in *G. macrophylla* and we showed that some of the latter type could be inadequate for transcript normalization under certain experimental conditions (Mafra et al., 2012). In summary, the optimal choice of internal controls for qPCR studies should be tailored to a particular species and be suitable for the particular experimental conditions that are under consideration.

## Author Contributions

YiH carried out the experimental design. YiH and YaH collected samples and helped on harvest. YaH and HY performed the experiments and analysis. YiH, WH, and ZW prepared the manuscript and coordinated its revision. All authors read and revised the manuscript, provided helpful discussions and approved its final version.

## Conflict of Interest Statement

The authors declare that the research was conducted in the absence of any commercial or financial relationships that could be construed as a potential conflict of interest.

## Footnotes

1 http://www.arabidopsis.org/index.jsp

2 http://www.ncbi.nlm.nih.gov/tools/primer-blast/

3 http://fulxie.0fees.us/?type=reference

4 http://www.gene-quantification.de/genex.html
